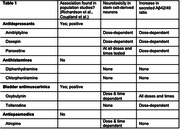# Differential Effects of Anticholinergic Medications in Patient hiPSC‐Derived Neurons

**DOI:** 10.1002/alz.092559

**Published:** 2025-01-03

**Authors:** Tiara A. Schwarze‐Taufiq, Inez Pranoto, Katherine Hui, Jordan Ogg, Ruowei Zhu, Paul K. Crane, Shelly L Gray, Jessica E. Young

**Affiliations:** ^1^ University of Washington, Seattle, WA USA; ^2^ Institute for Stem Cell and Regenerative Medicine, Seattle, WA USA; ^3^ Department of Medicine, University of Washington School of Medicine, Seattle, WA USA; ^4^ University of Washington School of Pharmacy, Seattle, WA USA

## Abstract

**Background:**

Anticholinergic (AC) use remains common in older adults despite evidence of safety risks, including dementia risk. Evidence from population studies suggests that dementia risk may vary by AC class. This variation might be explained by confounding by indication. *In vitro* models, using human neural cells where cells are directly experimentally exposed to a drug and phenotypic outcomes are measured, may address this issue. We identified classes of AC medications with and without an association with dementia based on population studies. We treated human induced pluripotent stem cell‐derived neurons (hiPSC‐Ns) with each AC medication and performed cellular assays to examine the effects on AD phenotypes.

**Method:**

We have generated hiPSC lines from participants in the Adult Changes in Thought Study (ACT), a longitudinal study in Seattle, WA. We differentiated hiPSC‐Ns from ACT cell lines using protocols in our laboratory. We treated neurons with different classes of AC medications: antidepressants (amitriptyline, doxepin, paroxetine), antihistamines (diphenhydramine, chlorpheniramine), bladder antimuscarinics (oxybutynin, tolterodine), and antispasmodics (atropine). As a control, we treated cells with the classic cholinergic compound carbachol. Cells were treated with two drug doses (10 uM, 50 uM) at two time points (24 hours, 48 hours). We used molecular assays to test AD‐associated cellular outcomes (neurotoxicity, synaptic gene expression and Amyloid beta [Aβ]).

**Result:**

We document differential effects of medications on cytotoxicity, secreted Aβ levels, and synaptic gene expression. Several drugs demonstrated dose‐dependent (doxepin, oxybutynin, paroxetine, amitriptyline) and/or time dependent (oxybutynin, paroxetine) neurotoxicity. We also observed differential effects on Aβ secretion. Oxybutynin, a bladder antimuscarinic, consistently induced an increase in the Aβ42/40 ratio, largely driven by increased secreted Aβ42. Further, all antidepressants and bladder antimuscarinics tested increased the secreted Aβ42/40 ratio at 50 uM. These findings are consistent across lines of different genetic backgrounds.

**Conclusion:**

Stem cell‐derived neuronal models provide a useful strategy to complement pharmacoepidemiological studies to help distinguish molecular pathways versus confounding by indication of drugs associated with dementia risk. Preliminary results suggest that different AC medications have differential effects on AD‐associated cellular outcomes that are consistent across hiPSC‐Ns derived from individuals with various genetic backgrounds, indicating a molecular effect of the drug.